# The genetic architecture of bone metastases: unveiling the role of epigenetic and genetic modifications in drug resistance

**DOI:** 10.20517/cdr.2025.28

**Published:** 2025-04-22

**Authors:** Ahmad Dawalibi, Mohamad Bakir, Khalid S. Mohammad

**Affiliations:** ^1^Department of Anatomy, College of Medicine, Alfaisal University, Riyadh 11533, Saudi Arabia.; ^2^Department of Medicine, College of Medicine, Alfaisal University, Riyadh 11533, Saudi Arabia.

**Keywords:** Bone metastases, drug resistance, genetic alterations, epigenetic modifications, tumor microenvironment

## Abstract

Bone metastases represent frequent and severe complications in various cancers, notably impacting prognosis and quality of life. This review article delves into the genetic and epigenetic mechanisms underpinning drug resistance in bone metastases, a key challenge in effective cancer treatment. The development of drug resistance in cancer can manifest as either intrinsic or acquired, with genetic heterogeneity playing a pivotal role. Intrinsic resistance is often due to pre-existing mutations, while acquired resistance evolves through genetic and epigenetic alterations during treatment. These alterations include mutations in driver genes like *TP53* and *RB1*, epigenetic modifications such as DNA methylation and histone changes, and pathway alterations, notably involving RANK-RANKL signaling and the PI3K/AKT/mTOR cascade. Recent studies underline the significance of the tumor microenvironment in fostering drug resistance, with components such as cancer-associated fibroblasts and hypoxia playing crucial roles. The interactions between metastatic cancer cells and the bone microenvironment facilitate survival and the proliferation of drug-resistant clones. This review highlights the necessity of understanding these complex interactions to develop targeted therapies that can overcome resistance and improve treatment outcomes. Current therapeutic strategies and future directions are discussed, emphasizing the integration of genomic profiling and targeted interventions in managing bone metastases. The evolving landscape of genetic research, including the application of next-generation sequencing and CRISPR technology, offers promising avenues for novel and more effective therapeutic strategies. This comprehensive exploration aims to provide insights into the molecular intricacies of drug resistance in bone metastases, paving the way for improved clinical management and patient care.

## INTRODUCTION

### Bone metastases: an overview

Bone metastasis is a common and severe consequence of several cancers, significantly affecting patient prognosis and quality of life^[1,2]^. Solid tumors, such as those originating from the breast, prostate, and lung, have a high propensity to metastasize to the bone^[1,3,4]^. Understanding the underlying genomic and genetic alterations associated with bone metastasis is crucial for developing targeted and effective treatment strategies.

The development of metastases results from a complex multistep process. Cells must disperse from the primary tumor, either individually or through collective migration, move into the circulatory or lymphatic systems as single cells or groups, infiltrate a capillary bed or extravasate into a distant organ, endure in a foreign microenvironment, and establish a secondary colony^[5-7]^.

Additional mechanisms may include perineural invasion, metastasis through ascites or cerebrospinal fluid, and intermediate metastases in the lymph nodes. Ultimately, disseminated cells may enter a state of inactivity, or their growth may be offset by apoptosis or immune system eradication, leading to latent metastasis that might only become clinically apparent decades after the excision of the primary tumor^[8]^. The survival and establishment of secondary colonies rely on various factors that intersect with essential stem-cell support pathways, growth factor signaling, positional and mechanical pathways, and inflammatory signaling^[8,9]^.

Although a variety of genes, molecules, and pathways have been identified as contributing to the effective execution of various metastatic processes, the regulation of phenotypic traits that enhance metastatic viability continues to be mostly unknown^[10]^. Metastases exhibit comparable mutational profiles to primary tumors, yet they possess driver alterations with greater frequency than primary tumors. Priestley *et al.* characterized 2,520 specimens of metastatic tumors from 22 solid tumor types. Other findings revealed no evidence of driver mutations unique to metastases; however, they identified alterations in the *MLK4* gene that are commonly linked to metastatic tumors^[11]^. Candidate driver variants were identified in over 98% of metastatic tumors, with 62% of patients possessing possibly therapeutically responsive variants^[11]^.

### Bone metastasis: the facts

Bone metastasis commonly presents with pain. The onset is usually slow, characterized by a dull, aching pain that intensifies at night. Bone metastases resulting in nerve root compression may manifest as radicular pain, distinct from mechanical pain. The vertebra is the predominant site of metastasis, and a significant consequence is spinal cord compression, classified as an oncologic emergency. Furthermore, bone metastases result in bone degradation, elevating the patient’s risk for atraumatic or imminent fractures. The presentation relies on the disease’s location; nonetheless, persistent discomfort is a common sign. Pathologic fractures of the thoracic and lumbar spine typically manifest with discomfort that exacerbates with sitting or standing, which leads to considerable morbidities due to pain, radiculopathy, deformities, and immobility^[12]^.

Moreover, osteolytic bone metastases account for 10%-30% of hypercalcemia instances in malignancy^[13]^. Osteolytic metastases involve increased osteoclastic bone resorption mediated by the RANK-RANKL signaling pathway, resulting in tumor cells releasing PTHrP, which stimulates osteoclasts and causes excessive bone resorption and hypercalcemia. Hypercalcemia symptoms encompass nausea, anorexia, stomach discomfort, constipation, and alterations in mental status. Prompt management of hypercalcemia necessitates hospitalization and intravenous hydration^[14]^.

The transcription factor c-Myb is integral to the development of bone metastases, especially in breast cancer. Research involving a highly metastatic subclone of MDA-MB-231 breast cancer cells (MDA-MB-231-BM) revealed that c-Myb is upregulated and plays a role in bone metastasis. Silencing of c-Myb in these cells resulted in a marked decrease in bone metastases in a murine model, as evidenced by diminished bioluminescent signals and a reduced number of metastatic nodules. This reduction was specifically not attributable to defects in cell proliferation, underscoring c-Myb’s distinct role in metastasis rather than tumor growth^[15]^. Subsequent investigations demonstrated that c-Myb facilitates metastasis by modulating mesenchymal markers, including Twist, Snail, Slug, and N-cadherin, which are linked to epithelial-to-mesenchymal transition (EMT) - a critical process for the invasion and dissemination of cancer cells. Furthermore, c-Myb engages with Wnt/β-catenin signaling, a pathway recognized for its role in promoting EMT and cancer advancement^[16]^.

In a separate experiment involving 4T1 breast cancer cells, the ablation of c-Myb did not influence primary tumor development but markedly diminished lung metastases, indicating its essential function in distant metastatic dissemination. c-Myb promotes bone metastasis by augmenting EMT and Wnt signaling, positioning it as a prospective therapeutic target for inhibiting metastatic advancement in breast cancer^[17]^.

MYB proto-oncogene like 2 (MYBL2) is a key regulator of prostate cancer (PCa) progression and bone metastasis. Bioinformatics analyses reveal its significant upregulation in metastatic PCa, correlating with poor prognosis. Elevated MYBL2 expression is associated with enhanced invasion and EMT, primarily through activation of the NOTCH3 signaling pathway. NOTCH3 silencing partially reverses MYBL2-induced invasion and EMT. *In vivo* studies confirm that MYBL2 overexpression promotes PCa proliferation and bone metastasis, highlighting its role in disease progression. Targeting the MYBL2/NOTCH3 axis may offer a promising therapeutic approach against metastatic PCa^[18]^.

Osteosarcoma (OS), a primary malignant bone tumor, shares several molecular pathways with bone metastases, which contribute to its aggressive metastatic behavior. The molecular mechanisms underlying the resemblance between OS and bone metastases are complex and multifaceted, involving various genetic, epigenetic, and microenvironmental factors. These mechanisms include alterations in signaling pathways, the chromatin landscape, and interactions between the tumor microenvironment (TME), collectively facilitating the metastatic potential of OS cells^[19]^.

## DRUG RESISTANCE: AN OVERVIEW

### Types of drug resistance: intrinsic and acquired

Drug resistance is categorized based on two popular types: de novo or intrinsic resistance, which is evident in patients who exhibit resistance from the onset of treatment due to inherent (mutation) resistance mechanisms, and acquired resistance, which develops in patients who gain resistance during therapy as a result of acquired resistance mechanisms (ARMs)^[20]^.

Acquired resistance can develop through the selection of clones harboring additional genetic or epigenetic changes that allow them to circumvent the effects of the therapeutic intervention or through the activation of compensatory pathways that render the cancer cells less susceptible to the targeted treatments^[11,21,22]^.

Genetic heterogeneity in cancer primarily arises from instability in the genome, leading to an increased mutation rate and subsequent cancer progression facilitated by various signaling pathways^[23,24]^. Inherited mutations (intrinsic resistance) often exist before or during early cancer treatment, facilitating adaptive responses. Acquired resistance arises from new mutations in subpopulations of cells that expand through selection within heterogeneous tumors. Studies have linked reactive oxygen species (ROS), genetic instability, and mutation induction, highlighting mitochondrial ROS’s role in chronic disease risk^[25]^. The mitochondrion is a pivotal regulator of metabolic-redox alterations in cancer cells that result in various gene modifications^[26]^. Alterations in mitochondrial metabolism, including diminished mitochondrial membrane potential and modifications in metabolic pathways, are associated with drug resistance in OS and other malignancies^[27]^.

### Importance of understanding molecular mechanisms driving resistance

Acquired resistance typically evolves in two phases^[28]^. The initial phase activates several cells that rapidly develop pre-resistance, leading to cellular reprogramming^[29,30]^. The subsequent phase occurs when the stable resistant phenotype proliferates, and the drug is ultimately administered. Pre-resistant cells are identified by the elevated expression of epidermal growth factor receptor (EGFR). Biomarkers, including EGFR, PDGFRB, and NRG1 in the patient’s blood, are elevated within one to four weeks after the administration of the appropriate medication. This is elucidated by an augmentation in transcriptional reprogramming toward those resistance markers. The pre-resistant cells exhibited limited expression of resistance markers^[31,32]^. Nonetheless, following the administration of the drug, the proportion of expressed resistance genes escalated by the conclusion of one week. The total resistance genes were activated to over 80% after several weeks of culture, indicating significant changes in expression patterns as the cells progressively developed resistance^[31,33]^.

Another resistance mechanism pertains to pathway redundancy and oncogenic bypass concerning targeted agents. The oncogene skip mechanism is illustrated by the activation of an alternative receptor tyrosine kinase (RTK) that induces resistance to the primary tyrosine kinase inhibitor (TKI)^[34]^. The simultaneous activation of the bone morphogenetic protein-signaling pathway was identified as a cause of EGFR-TKI-resistant lung squamous cancers^[35]^. Bypass resistance processes can also be attained through the modification of feedback cycles^[36]^.

Resistance can also develop in a “pathway-independent” manner via methods including EMT, TME disruption, and angiogenesis modifications. The activation of AXL receptor tyrosine kinase (AXL) and the concomitant EMT engender resistance to EGFR-targeting therapy in non-small cell lung carcinoma (NSCLC)^[37]^.

Mesenchymal-epithelial transition (MET) enhancement often induces resistance to anti-EGFR therapies like erlotinib and gefitinib used in lung cancer treatment. This acquired resistance is reversible due to dynamic phenotypic and genotypic expressions in heterogeneous tumors, where resistance diminishes without drug exposure. Evidence from biopsy analyses in lung cancer shows that phenotypic and genotypic alterations progressively evolve into cell mutations, responding predictably to targeted therapy^[28,38-40]^.

### Relevance of genetic and genomic alterations in drug resistance

Key genetic factors associated with drug resistance include secondary mutations that reactivate target sites, activation of alternative signaling pathways, and epigenetic modifications. Molecular alterations, such as mutations or gene expression loss, can drive acquired resistance. For example, the G2032R mutation in the ROS1 kinase domain confers resistance to Crizotinib in lung adenocarcinoma (LADC) by disrupting its inhibition of ALK, ROS1, and MET tyrosine kinases^[41]^. A secondary mutation in the ectodomain of the EGFR, specifically S492R, conferred resistance to cetuximab in colorectal cancer by obstructing the binding of the EGFR antibody to its target^[42]^.

Genomic alterations that disrupt signaling proteins functioning upstream or downstream of a therapeutic target can also lead to acquired resistance. In EGFR-mutant cancer cells, an oncogenic mutation in the catalytic subunit alpha of phosphatidylinositol-4,5-bisphosphate 3-kinase (PIK3CA) was adequate to induce resistance to gefitinib, an EGFR inhibitor. Comparable mutations have also been identified in EGFR-mutant tumor specimens exhibiting acquired resistance to erlotinib, another EGFR inhibitor^[43,44]^.

KLF5 proteins experience various posttranslational modifications that regulate their protein levels or transactivation activities. These modifications encompass phosphorylation, acetylation, ubiquitination, and sumoylation. KLF5 phosphorylation enhances its activity, whereas KLF5 ubiquitination diminishes its protein levels; the roles of acetylation and sumoylation are contingent upon context^[45]^. KLF5 is known to undergo acetylation by p300 and deacetylation by HDAC1 and SET. Reports indicate that the acetyltransferase p300 acetylates KLF5 at K369, seemingly augmenting the transactivation activity of KLF5. An independent study corroborated the notion: utilizing a KLF5 K369 acetylation-specific antibody, it was demonstrated that TGFβ recruits p300 to acetylate KLF5. The SET histone chaperone was demonstrated to inhibit KLF5’s DNA binding and cell proliferation functions, which correlates with a reduction in KLF5 acetylation. HDAC1, a deacetylase, can interact with KLF5 to impede its binding to DNA and KLF5-mediated promoter activation by inhibiting the acetylation of KLF5 by p300^[46,47]^.

A study using PCa cells with the KLF5K369Q mutation (mimicking Ac-KLF) found that nitazoxanide (NTZ) effectively inhibited invasion and bone metastasis in both preventive and therapeutic settings. NTZ reduced KLF5K369Q-induced gene regulation, particularly MYBL2, which enhances bone metastasis. NTZ also decreased KLF5K369Q binding to the MYBL2 promoter and impeded osteoclast differentiation^[48]^. In advanced PCa, bone-derived TGF-β induces KLF5 acetylation (Ac-KLF5), promoting osteoclastogenesis and bone metastasis by activating CXCR4, leading to IL-11 secretion and SHH/IL-6 signaling. Ac-KLF5 maintains the mesenchymal phenotype, promotes tumor growth, and induces docetaxel resistance, which can be mitigated by targeting CXCR4 with plerixafor. These findings highlight the potential of inhibiting Ac-KLF5/CXCR4 signaling to treat chemoresistant bone metastasis in PCa^[49]^.

Drug resistance in cancer can arise at various stages, including before metastasis, during metastatic progression, or due to interactions with the bone microenvironment after metastasis. Cancer cells may possess genetic mutations that confer resistance even before metastasis, with genomic instability promoting resistant clones that survive initial treatments and drive metastatic spread, emphasizing the importance of early genomic analysis for effective therapy customization^[50]^. During metastatic progression, stressful conditions may favor the emergence of resistant phenotypes^[51]^. Additionally, the bone microenvironment plays a critical role in inducing drug resistance through interactions between tumor cells and bone marrow stromal cells, which activate stress response pathways, autophagy, and transcriptional changes that contribute to a drug-resistant phenotype^[52]^.

The exact timing and mechanisms of resistance acquisition remain unclear, underscoring the need for further research to improve therapeutic strategies.

## GENETIC ALTERATIONS IN DRUG RESISTANCE

### Mutations in driver genes

Inherent drug resistance generally manifests at the onset of drug therapy and is referred to as primary resistance. Immediate resistance arises when the targets of drug therapy, specifically oncogenic drivers or signaling pathways (such as KRAS or EGFR mutations), are not inhibited or fail to respond to the medication, thereby triggering significant resistance to targeted therapy^[53]^.

**Figure 1:**
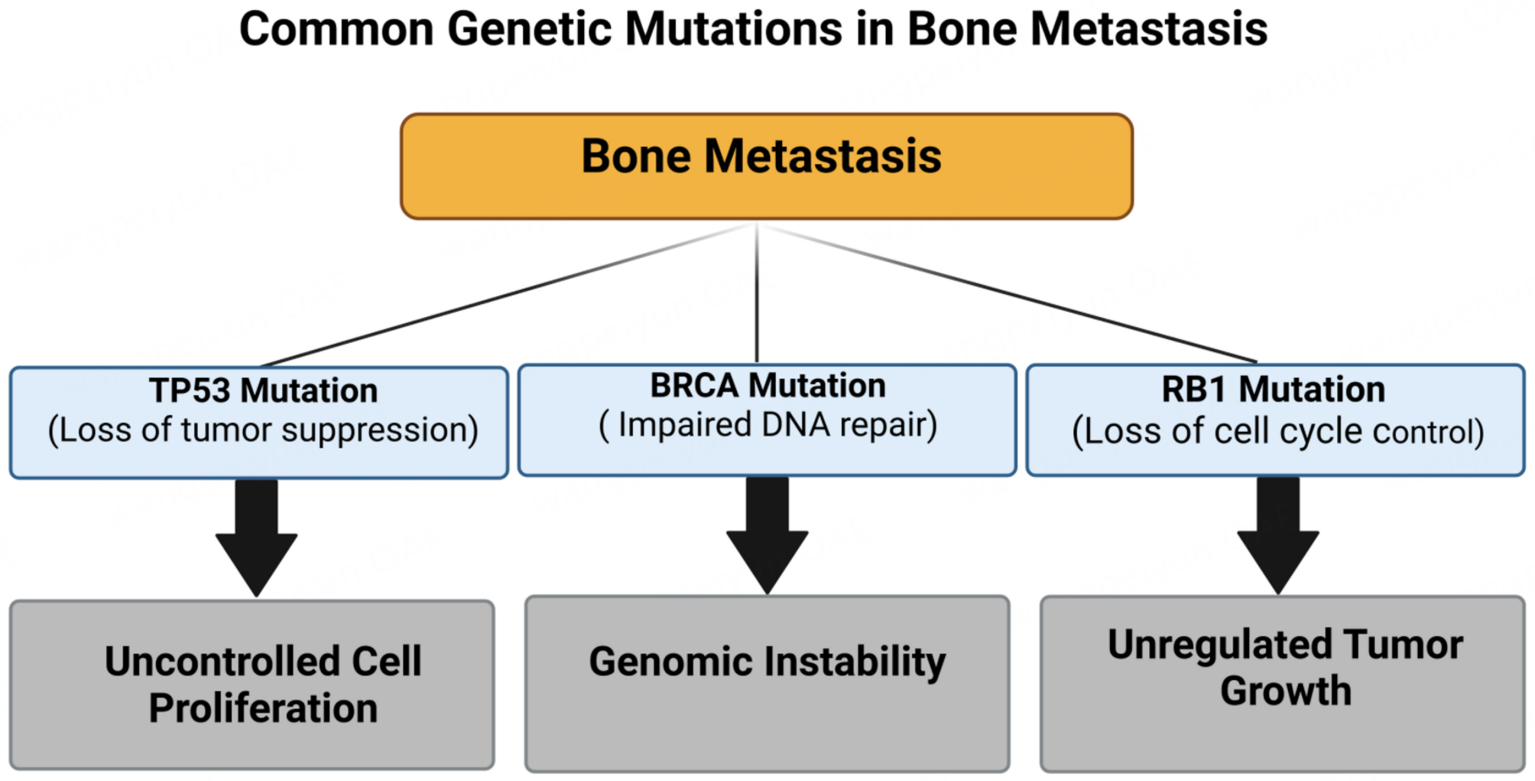
This schematic illustrates three major genetic mutations commonly observed in bone-metastatic cancers - TP53, BRCA, and RB1 - and their functional consequences. TP53 mutations lead to a loss of tumor-suppressor activity, promoting unchecked cell proliferation. BRCA mutations compromise DNA repair pathways, driving genomic instability. RB1 mutations disrupt cell cycle regulation, resulting in unregulated tumor growth. Together, these genetic alterations foster an environment conducive to the development and progression of bone metastases. Created in BioRender. Mohammad, K. (2025) https://BioRender.com/w2dhbh8.

**Figure 2:**
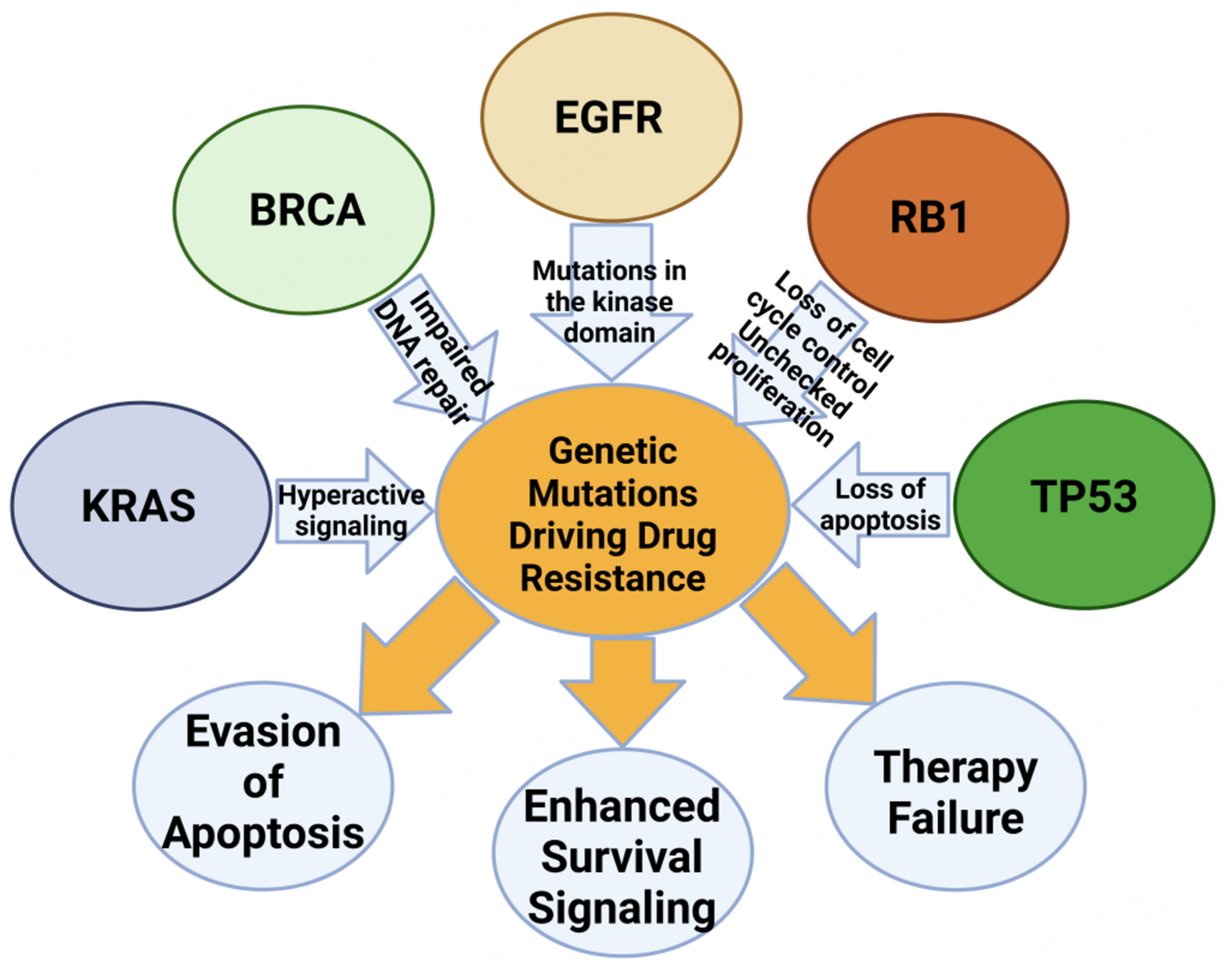
Genetic mutations and their role in drug resistance and cancer progression. This illustration depicts the intricate network of genetic mutations that underpin drug resistance and cancer progression. Mutations in the *BRCA* gene, represented by the red circle, impair DNA repair mechanisms, increasing susceptibility to further genetic aberrations that may lead to cancer. Similarly, alterations in the *KRAS* gene, shown in green, trigger hyperactive signaling pathways that foster cancer cell growth and survival. The purple oval indicates mutations in the EGFR, specifically in the kinase domain, which activate downstream signaling pathways independently of ligand binding, thereby promoting unregulated cell proliferation. The magenta oval represents the loss of function in the *RB1* gene, leading to disrupted cell cycle control and unchecked cell proliferation. Additionally, mutations in the *TP53* gene, shown in blue, result in the loss of apoptosis, allowing cells with damaged DNA to survive. These genetic alterations collectively enhance survival signaling, evade apoptosis, and facilitate unchecked cell proliferation, culminating in therapy failure, as depicted by the grey circles. This figure highlights how these mutations collectively contribute to the complexity of drug resistance in cancer therapy. Created in BioRender. Mohammad, K. (2025) https://BioRender.com/86cygfh. EGFR: Epidermal growth factor receptor.

Mutations in critical genes such as *TP53*, *BRCA*, and *RB1* have been identified in multiple malignancies, including those that metastasize to bone [Figure 1]. In OS, a kind of bone cancer, mutations in TP53 and RB1 are prevalent. A study indicated that 47% of OS cases exhibited mutations in TP53 or RB1^[54]^. Furthermore, studies indicate that the concurrent deletion of TP53 and RB1 in murine osteoblasts results in a high incidence of metastatic OS^[55]^. Although BRCA mutations are predominantly linked to breast and ovarian cancers, they may also contribute to other malignancies that can metastasize to bone. A case study of castration-resistant prostate cancer (CRPC) with bone metastases demonstrated biallelic loss of RB1 and BRCA2, together with a truncating mutation in TP53^[56]^. The results indicate that mutations in TP53, BRCA, and RB1 correlate with bone metastases in specific cancer types. Thoenen *et al.* emphasize the significant involvement of TP53 and RB1 mutations in the onset and advancement of OS, especially in facilitating bone metastases^[55]^. In a murine model, researchers exhibited that the concurrent ablation of TP53 and RB1 in osteoblasts markedly elevated the probability of aggressive and metastatic OS [Figure 2].

Mutations in the TP53 tumor suppressor gene hinder cellular repair systems and facilitate unregulated growth. The loss of RB1 impairs cell cycle regulation, hence increasing tumor aggressiveness. The interplay of these two modifications enhances metastatic capability, especially in the bone microenvironment. This work demonstrates a significant causal relationship between genetic modifications in TP53 and RB1 and their involvement in bone metastases^[55]^.

Studies have identified biallelic loss of RB1 and BRCA2, along with a truncating TP53 mutation, in CRPC prone to bone metastasis. RB1 loss is associated with treatment resistance and disease progression, while BRCA2 mutations compromise DNA repair, promoting mutations and metastasis. TP53 mutations further enhance genomic instability by impairing apoptosis. These findings highlight the combined impact of TP53, BRCA2, and RB1 mutations in promoting bone metastasis in PCa^[56]^.

A recent study presented an extensive genomic analysis of OS, highlighting recurrent mutations in TP53 and RB1 as the predominant changes^[54]^. The research indicated that about 47% of patients exhibited gene mutations, reinforcing their carcinogenesis and bone metastasis involvement. TP53 mutations weaken tumor suppression, enabling uncontrolled cell growth, while RB1 mutations promote cell cycle progression and metastasis. Their high prevalence underscores their critical role in OS’s metastatic potential, making them key genetic targets for understanding and treating bone metastases^[54]^.

Several key mutations that are commonly observed in bone metastases have been identified. Mutations in the tumor suppressor gene *TP53* are frequently observed in bone metastases, as they play a crucial role in the development and progression of various cancers^[22]^. Mutations in the *BRCA* genes, which are involved in DNA repair, have also been implicated in developing bone metastases^[11]^. Additionally, alterations in the *RB1* gene, which is involved in cell cycle regulation, have been linked to the development of bone metastases.

Mutations activating the RAS/mitogen-activated protein kinase (MAPK) pathway (e.g., in KRAS, NRAS, and BRAF) are linked to increased osteoclast activity and bone resorption, particularly in conditions like Noonan, Costello, and cardiofaciocutaneous syndromes. However, their direct role in metastatic bone resorption remains under investigation^[57]^.

While these mutations are known to activate the RAS/MAPK pathway involved in cell proliferation and differentiation, their specific impact on osteoclast activity in metastatic settings requires further research^[57]^.

Evidence linking these mutations to drug resistance in bone malignancies, particularly OS, remains limited. Most studies focus on other cancers like colorectal cancer, where these mutations are prevalent. Unlike other malignancies, primary bone tumors (OS) and bone metastases present a distinct landscape. Research has identified oncogenic mutations in *BRAF*, *KRAS*, and *NRAS* genes in the bone marrow and plasma cell-free DNA of multiple myeloma patients, suggesting their presence in bone-related cancers. However, their role in drug resistance in primary bone tumors like OS remains unclear^[58]^.

Although well-documented in other cancers, the relevance of KRAS, NRAS, and BRAF mutations to drug resistance in bone tumors requires further investigation.

### Epigenetic modifications

Epigenetic alterations, such as DNA methylation and histone changes, significantly contribute to medication resistance^[59]^ [Table 1].

**Table 1:** Summary of epigenetic modifications and their roles in drug resistance in bone metastases

**Epigenetic modification**	**Associated molecular mechanism**	**Impact on drug resistance**	**Cancer type (if specified)**
DNA methylation	Silencing of tumor suppressor genes, oncogene activation	Promotes adaptive resistance, decreased apoptosis sensitivity	Solid tumors, including OS
Histone acetylation/deacetylation	Regulation of gene transcription, chromatin remodeling	Modulation of gene expression related to drug sensitivity, reversible resistance to targeted therapies	Lung cancer, PCa
Histone methylation/demethylation	Alterations in chromatin state and transcriptional regulation	Promotes cellular plasticity, adaptation to therapy pressures, altered response to chemotherapeutic agents	Various metastatic cancers
Histone modification (KMT2C mutations)	Disrupted histone modification and gene expression	Increased metastatic potential and poor response to therapies	OS
MiRNA-mediated epigenetic regulation	Regulation of gene expression post-transcriptionally (miR-181c targeting OPN)	Increased or decreased drug sensitivity, particularly via modulation of apoptosis and EMT pathways	Breast cancer
Epigenetic regulation of EMT	Chromatin remodeling influencing EMT gene expression	Enhanced invasiveness, metastatic spread, and acquired drug resistance, reversible upon epigenetic targeting	Breast and PCa
Dynamic epigenetic state	Reversible epigenetic transitions facilitating rapid adaptability	Temporary resistance to targeted therapies (e.g., EGFR inhibitors)	Lung cancer

OS: Osteosarcoma; PCa: prostate cancer; OPN: osteopontin; EMT: epithelial-to-mesenchymal transition; EGFR: epidermal growth factor receptor.

These alterations may result in the repression of tumor suppressor genes or the activation of oncogenes, hence facilitating therapeutic resistance^[60,61]^. The dynamic nature of epigenetic modifications facilitates fast adaptability to therapeutic forces, hence complicating treatment^[60]^. The complex nature of genetic changes in bone metastases is highlighted by the discovery of notable somatic mutations associated with treatment resistance, especially in aggressive malignancies like OS. Specific mutations, particularly in KMT2C, which is vital for histone modification, are significantly increased in patients who develop metastases and demonstrate inadequate responses to treatment^[62]^. Moreover, recent evidence validates the efficacy of circulating tumor DNA (ctDNA) as a non-invasive approach for identifying treatable mutations in advanced cancers, thus elucidating mechanisms of therapy resistance^[22]^. The complex interplay of genomic and genetic alterations, as well as epigenetic modifications, contribute to the development and progression of bone metastases. Understanding these mechanisms is crucial for the development of targeted therapies and improved patient outcomes^[11,21,63,64]^.

Epigenetic modifications contribute to acquired resistance to targeted therapies and chemotherapies, as significant epigenetic alterations in tumors can produce varied gene expression patterns to adapt to pharmacological treatment. For example, if the lung cancer cell line PC9 was exposed to an EGFR inhibitor, a subset of the typically susceptible cells developed resistance by adopting a seemingly reversible epigenetic state, which could be mitigated by the administration of a histone deacetylase inhibitor^[65]^. Moreover, the integration of DNA methylation and RNA expression profiling identified potential DNA methylation drivers of acquired cisplatin resistance across multiple types of cancer^[66,67]^. Considering that epigenetic modulators can facilitate the resensitization of tumors to chemotherapies, numerous clinical trials of epigenetic therapies are currently in progress for solid tumors as agents to modulate drug resistance^[68-71]^.

### Tumor heterogeneity

Tumor heterogeneity, a defining feature of advanced cancers, drives drug resistance through genetic alterations that activate survival pathways or alternative signaling cascades, enabling cells to evade treatment^[11,21,22]^.

Despite extensive sequencing, unique driver gene mutations exclusive to metastases have not been identified. Instead, genetic differences between primary and metastatic sites are often inconsistent and can also appear in primary tumors under varying conditions^[72-77]^.

Mathematical modeling of untreated patients revealed that primary tumors and their metastases share identical driver mutations. Reiter *et al.* found that all metastases in each patient harbored the same functional driver mutations, with inter-metastatic heterogeneity being largely nonfunctional^[78]^.

A study of 617 metastatic breast cancer patients showed greater genetic complexity, higher mutational burden, and increased clonal diversity compared to early-stage cancers^[79]^.

Furthermore, prevalent germline variants of the *APOE* gene have been demonstrated to correlate with varying outcomes in melanoma^[80]^.

Turajlic *et al.* found that in renal cancer, 9p deletion - affecting CDKN2A and CDKN2B tumor suppressor genes - was the only alteration significantly associated with metastasis. Primary tumors with PBRM1-SETD2 and PBRM1-PI3K mutations and high heterogeneity were linked to reduced progression, suggesting metastatic potential arises from genetic alterations already present in primary tumors^[72]^.

### Specific examples in bone metastases

KRAS mutation is the most common genetic alteration in LADC in Western populations and is linked to poor outcomes in bone-metastatic cases. Currently, no standard treatment guidelines exist for these patients. A retrospective study of 134 LADC patients with bone metastases assessed the impact of KRAS status on bisphosphonate (BTx) and radiation therapy (RTx). KRAS mutations were found in 30.6% of patients and associated with significantly reduced median overall survival (5.1 *vs.* 10.2 months; *P* = 0.008). While BTx and RTx improved overall survival regardless of KRAS status, their benefits were statistically significant only in KRAS wild-type patients (*P* = 0.032 and *P* = 0.031, respectively). These findings highlight KRAS mutation as a negative prognostic marker and underscore the importance of KRAS status in guiding therapy for bone-metastatic LADC^[81]^.

The efficacy of combining bisphosphonates with EGFR-TKIs in EGFR-mutant NSCLC patients with bone metastases (BM) remains unclear. A study involving 1,560 NSCLC patients with BM identified 356 with EGFR mutations. Of these, 91 received EGFR-TKIs alone, while 105 received EGFR-TKIs along with bisphosphonates. The combination therapy significantly improved progression-free survival (PFS: 11.6 *vs.* 9.3 months; HR = 0.68, *P* = 0.009), while overall survival remained similar (OS: 20.5 *vs.* 19.5 months; HR = 0.95, *P* = 0.743). Multivariate analysis confirmed that EGFR mutation is an independent prognostic factor for OS (HR = 0.710, *P* = 0.021). These findings suggest that bisphosphonates may enhance the efficacy of EGFR-TKIs in EGFR-mutant NSCLC with BM^[82]^.

Drug resistance is linked to HER2 mutations in bone metastases of breast cancer, especially in estrogen receptor (ER)-positive breast cancer. These mutations may stimulate the HER2 signaling pathway, leading to resistance to conventional treatments. HER2 mutations occur in around 3% of bone metastases originating from breast cancer, with elevated frequencies observed in the pleomorphic subtype of lobular carcinoma. These mutations frequently manifest in HER2-negative instances identified through standard testing, potentially overlooking chances for targeted therapy^[83]^. In ER-positive metastatic breast cancer, acquired HER2 mutations induce resistance to ER-targeted therapy, including aromatase inhibitors, fulvestrant and tamoxifen. These mutations confer estrogen independence and resistance, which can be mitigated by integrating ER-targeted therapy with HER2 kinase inhibitors such as neratinib^[84]^. HER2 mutations may provide resistance to reversible HER2 inhibitors like lapatinib, although they retain sensitivity to irreversible inhibitors such as neratinib^[85]^. Further resistance pathways encompass the development of mutations such as NF2, which may complicate therapy with HER2-targeted medicines. The combination of HER2 and ERK inhibitors may assist in surmounting this resistance^[86]^. HER2 mutations in breast cancer, especially in metastatic contexts, indicate that these alterations are crucial contributors to carcinogenesis and therapeutic resistance. This underscores the necessity for focused medicines that specifically address these alterations^[87,88]^. Novel therapy techniques are under investigation, encompassing dual-blockade methodologies and combinations with other drugs such as CDK4/6 inhibitors and immune checkpoint inhibitors, to enhance outcomes for patients with HER2-positive breast cancer^[89,90]^.

The complex interplay of genomic and genetic alterations strongly influences drug resistance in bone metastases and OS. Recent studies have shown that somatic variations in critical genes, such as KMT2C, reveal the mechanisms driving therapeutic resistance - underscoring the urgent need for personalized treatment strategies tailored to each individual’s genetic profile^[64]^. In a case study of metastatic salivary acinic cell carcinoma, mutations including deletions in CDKN2A and CDKN2B, as well as truncating mutations in ARID2, were associated with tumor aggressiveness and resistance to traditional therapy^[91]^. These findings support the implementation of targeted medicines that correspond with patients’ specific genomic alterations, thereby improving survival rates and overcoming the traditional constraints of cytotoxic treatments in managing bone metastases.

## GENOMIC ALTERATIONS AND PATHWAYS IN DRUG RESISTANCE

### Copy number variations

Chromosomal Aberrations: Structural chromosomal alterations, such as amplifications (e.g., oncogene MYC overexpression) and deletions (e.g., loss of tumor suppressor PTEN), provide cancer cells with a selective advantage. These changes, including translocations and copy number variations (CNVs), can affect metastasis-related genes. For example, CNVs may upregulate RANKL, promoting osteoclast activation and bone resorption in bone metastases. Understanding how metastatic cells alter their genetic structure is essential, as these aberrations are often selected to enhance tumor proliferation, angiogenesis, and bone remodeling within the bone microenvironment^[10]^.

### Chromosomal instability

Aneuploidy, defined by irregular chromosome counts, is commonly detected in metastatic bone cancers. This genetic modification fosters heterogeneity in the tumor, facilitating clonal development and picking out resistant tumor cell types specifically inside the bone microenvironment^[92]^. Structural chromosomal alterations, including translocations, deletions, and amplifications, frequently encompass genes essential to metastasis. Enhancement of the MYC oncogene or deletions of the RB1 tumor suppressor gene correlate with increased metastatic potential and survival in bone-specific settings^[93]^.

Interactions within the bone microenvironment reveal that chromosomal instability increases tumor cell plasticity, facilitating adaptation to this environment. This involves the alteration of essential signaling pathways such as RANK/RANKL and Wnt, which control osteoclast and osteoblast function, thereby promoting metastatic colonization^[92]^.

Therapeutic resistance arises from genomic alterations associated with chromosomal instability, which activate survival pathways and escape drug-induced apoptosis^[94]^. This results in resistance to therapies such as bisphosphonates and denosumab, which are frequently utilized for bone metastases.

### Gene expression profiles

The differential expression of cytochrome P450 (CYP) enzymes, particularly CYP3A4, CYP3A5, and CYP2C8, significantly contributes to drug resistance in bone metastasis. These enzymes metabolize various anticancer agents, including taxanes like docetaxel and paclitaxel, and their expression within tumor cells, influenced by the bone microenvironment, can alter drug efficacy. Localized drug metabolism resulting from this expression reduces therapeutic effectiveness and promotes resistance^[95]^.

CYPs are present in the bone marrow microenvironment, particularly in mesenchymal stromal cells, and may contribute to drug resistance in metastatic solid tumors. These enzymes support hematopoietic stem cell maintenance and protect myeloma and leukemia cells from toxicity. Similar mechanisms are thought to apply to solid tumors in bone, where variable CYP expression may influence drug resistance^[96]^.

Strategies to overcome CYP-mediated drug resistance include developing CYP inhibitors, prodrugs, and analogs to reduce the rapid metabolism of anticancer drugs by CYP enzymes. A study highlights that drug-metabolizing enzymes (DMEs), particularly CYP1B1, contribute to chemoresistance by metabolizing anticancer agents, reducing their efficacy. While not directly addressing bone metastasis, it emphasizes the role of DMEs in pharmacokinetic resistance. Understanding DME expression, especially within the CYP family, may clarify their contribution to drug resistance in various cancers, including bone metastasis^[97]^.

Inhibiting CYP3A4-mediated metabolism with protease inhibitors has been proposed as a means to enhance the efficacy of taxane-based therapies. While CYP enzymes play a well-established role in drug resistance, other factors, such as efflux transporters and genetic mutations, also contribute. Understanding the interplay among these mechanisms is vital for developing effective treatments against bone metastasis^[95]^.

### Dysregulation of signaling pathways

Dysregulation of the mTOR pathway correlates with heightened tumor heterogeneity and drug resistance, especially in PCa bone metastasis. Inhibiting this pathway with agents such as Rapalink-1 demonstrates the potential in surmounting resistance^[98]^. Bone sarcomas and other malignancies show drug-resistant phenotypes in cancer stem cells (CSCs) resulting from pro-stemness signaling pathways including Wnt/β-Catenin, NOTCH, and JAK/STAT. Together with metabolic and epigenetic alterations, these pathways help to explain drug resistance^[99,100]^.

AXL is not essential for sustaining intrinsic resistance, and a transcriptional cell state differentiation associated with low levels of microphthalmia-associated transcription factor (MITF) and elevated levels of nuclear factor of kappa light polypeptide gene enhancer in B cell (NF-κB) may influence melanoma resistance to MAPK pathway inhibitors^[101]^. Furthermore, the TME may shield cancer cells from cytotoxic agents, enabling the development of acquired resistance and resulting in disease recurrence. The TME induces innate resistance to RAF inhibitors via the secretion of hepatocyte growth factor (HGF)^[102]^.

The PI3K/AKT/mTOR signaling pathway is essential for facilitating cell survival, proliferation, and metabolism, and its improper regulation has been associated with medication resistance in multiple malignancies^[103]^. PIK3CA mutations enhance *in vitro* PI3K activity, and the expression of p110α mutants in cells induces AKT activation without growth factors stimuli, subsequently resulting in cancer formation. Recent studies indicate that somatic mutations in PIK3CA are prevalent in several human malignancies, including breast, colon, and endometrial cancers, as well as glioblastoma^[104,105]^. Numerous genetic modifications are recognized to activate the PI3K-AKT signaling pathway; however, the second most prevalent genetic anomaly observed in human malignancies is the inactivation of the PTEN tumor suppressor gene^[103]^. Knuefermann *et al.* showed that breast cancer cell lines co-expressing HER2 and HER3 had elevated AKT phosphorylation levels and were linked to enhanced resistance to several chemotherapeutic drugs^[106]^. The selective suppression of PI3K or AKT activity induces apoptosis; thus, the postulated mechanism for this synergy is the enhancement of apoptosis.

The MAPK/ERK pathway regulates cell growth and development, and its dysregulation in bone sarcomas promotes growth, invasion, metastasis, and treatment resistance. Clinical trials show the benefits of MAPK/ERK-targeted therapy in patients with unresectable or metastatic OSs. ERK proteins, part of the serine/threonine kinase family, are activated by tyrosine phosphorylation in response to growth hormones like insulin and NGF, mediating inflammatory and carcinogenic signals. The MAPK cascade transmits, amplifies, and integrates signals from the cell membrane to the nucleus, influencing processes such as growth, differentiation, inflammation, and apoptosis^[107]^. The MAPK pathway, a crucial cellular control and maintenance signaling sequence, is highly significant in bone metastases. The activation of this pathway by IL-6 greatly enhances cell migration and infiltration^[108]^. Research has shown that modifying the MAPK pathway significantly influences the survival and proliferation of tumor cells, and it also impacts how they interact in the bone microenvironment, hence altering the development and progression of bone metastases^[109]^.

The Wnt/β-catenin signaling pathway plays a key role in osteogenesis during breast cancer bone metastasis by modulating the bone microenvironment. A study using a 3D *in vitro* model with nano clay-based scaffolds, osteogenically differentiated MSCs, and breast cancer cells (MCF-7, MDA-MB-231) showed increased expression of Wnt components (Wnt-5a, β-catenin, AXIN2, LRP5) in MSCs cultured with MCF-7 compared to MDA-MB-231. Elevated β-catenin protein levels were observed in MCF-7 co-cultures. ET-1-induced activation of Wnt/β-catenin signaling enhanced bone formation, while DKK-1 inhibition reduced bone growth, replicating bone lesions seen in breast cancer^[110]^.

## ROLE OF THE BONE MICROENVIRONMENT IN DRUG RESISTANCE

### Interaction between tumor cells and bone cells

The bone microenvironment, particularly bone marrow adipocytes, plays a critical role in drug resistance by stimulating cancer-associated fibroblasts (CAFs) and immune evasion, promoting cancer progression and resistance^[111]^. Osteopontin (OPN) is a glycophosphoprotein that enhances tumor progression and drug resistance in bone metastasis through its interaction with integrins and receptors, thereby stimulating signaling pathways that boost survivability and resistance^[112]^. Chemotherapy resistance is a major challenge in breast cancer treatment, with evidence indicating that microRNAs (miRNAs) contribute to resistance development. Notably, miR-181c is under-expressed in the chemoresistant MCF-7/ADR cell line compared to wild-type MCF-7 cells. Overexpressing miR-181c reduced cell proliferation, reversed doxorubicin resistance, and decreased tumor formation *in vitro* and *in vivo*. Bioinformatics analysis identified OPN as a direct target of miR-181c; its downregulation enhances p53-dependent apoptosis, improving chemosensitivity^[113]^. Genetic instability and variability, frequently influenced by anti-apoptotic proteins such as Bcl-xL, play a significant role in organ-specific chemoresistance in bone metastasis^[114]^.

Bone metastases are particularly challenging in advanced breast cancer due to their complex genetic basis. RICTOR mutations, found in 5% of bone-metastatic breast cancer samples, suggest its role in metastatic cell adaptation to bone^[115]^.

Additionally, genes such as SMARCA4 and AURKA are linked to enhanced cell invasiveness and poor survival outcomes, highlighting their potential roles in metastasis, including bone-specific spread^[115,116]^.

These findings align with the seed and soil hypothesis, which posits that metastatic tumor cells exhibit a preference for specific organ environments. The higher mutational frequency of RICTOR in bone metastases underscores the plasticity and adaptability of metastatic cells, further supporting their predilection for bone as a metastatic site. Understanding these genetic drivers provides a foundation for developing targeted therapies to manage bone metastases more effectively^[115,117]^.

Osteoclasts drive bone resorption, releasing growth mediators like TGF-β, IGF, and vascular endothelial growth factor (VEGF), which promote tumor growth and drug resistance. Cytokines IL-6 and IL-11 activate osteoblasts, releasing matrix components and growth factors that enhance tumor survival, especially in osteoblastic lesions. IL-6 also promotes osteoclastogenesis by increasing RANKL expression on osteoblasts or stromal cells, favoring bone resorption in osteolytic lesions. These interactions between cytokines, osteoblasts, osteoclasts, and tumor cells highlight the complex role of the bone microenvironment in tumor progression and therapeutic resistance^[108]^. The bone metastatic microenvironment exhibits an imbalance between osteoclast-driven bone resorption and osteoblast-driven bone creation, resulting in enhanced turnover of bone and metastasis. Utilizing Bisphosphonates and RANKL inhibitors such as Denosumab to target osteoclast function has demonstrated potential in managing bone metastases^[118]^. These medicines seek to decrease bone resorption and minimize the influence of the bone microenvironment on tumor advancement and chemotherapy resistance. Nonetheless, addressing the complicated relationships among osteoblasts, osteoclasts, and tumor cells may necessitate combinations of treatments that focus on both the tumor cells and the bone microenvironment.

### Stromal and immune cell contributions

CAFs contribute to drug resistance in bone metastasis by modulating the TME. By releasing soluble factors such as proteins, cytokines, extracellular vesicles, and metabolites, CAFs promote tumor progression, induce genetic changes in cancer cells, and enhance their survival and resistance to therapy^[119-121]^. CAFs affect genetic pathways that contribute to drug resistance. They can initiate EMT, activate survival pathways, and enhance stemness-related programs in tumor cells, which are essential for therapeutic resistance^[122,123]^.

CAFs are heterogeneous, with various subtypes demonstrating differing roles in drug resistance. Specific subtypes secrete immunosuppressive and stem cell-promoting factors, which enhance resistance to therapies, including immunotherapy^[124,125]^. Additionally, CAFs play a role in the remodeling of the extracellular matrix (ECM) and facilitate cancer cell invasion and metastasis. Bone metastasis is notably influenced by CAFs, which promote the dissemination and colonization of cancer cells within bone tissue^[126]^.

CAFs also enhance the resistance of cancer cells to chemotherapeutic agents, resulting in accelerated proliferation and possible recurrence. Therefore, targeting CAFs represents a promising therapeutic approach due to their involvement in drug resistance. Anti-CAF therapies seek to undermine the supportive function of CAFs within the TME, potentially reducing drug resistance and enhancing treatment efficacy^[127,128]^.

### Hypoxia and angiogenesis

The hypoxic bone marrow microenvironment promotes bone metastasis, with HIF-1α, a key hypoxia marker, linked to poor prognosis and resistance to radio- and chemotherapy in breast cancer. Its expression and activity, mediated by interactions with Wwox and TAZ from the Hippo pathway, remain poorly understood. In bone metastasis specimens, HIF-1α and TAZ co-localized in neoplastic and supportive cells, with elevated nuclear expression under hypoxia. TAZ regulated HIF-1 activity, enhancing metastasis-stroma interactions. Blocking cyclooxygenase-2 with NS398 reduced HIF-1α, activated upstream Hippo pathway mechanisms, and impaired bone-metastatic cell proliferation by promoting TAZ degradation^[129]^.

In OS, hypoxia-induced HIF-1α activation increases CXCR4 expression, promoting metastasis, while deubiquitinases like HAUSP stabilize HIF-1α, driving processes such as EMT^[130,131]^.

Focusing on the hypoxic TME and HIF-1α-associated pathways presents promising therapeutic approaches. The inhibition of the HIF-1α/CXCR4 axis may be utilized for innovative therapies. Furthermore, compounds such as Oroxylin A exhibit potential in reversing hypoxia-induced drug resistance through the inhibition of HIF-1α-mediated transcription^[132]^.

**Figure 3:**
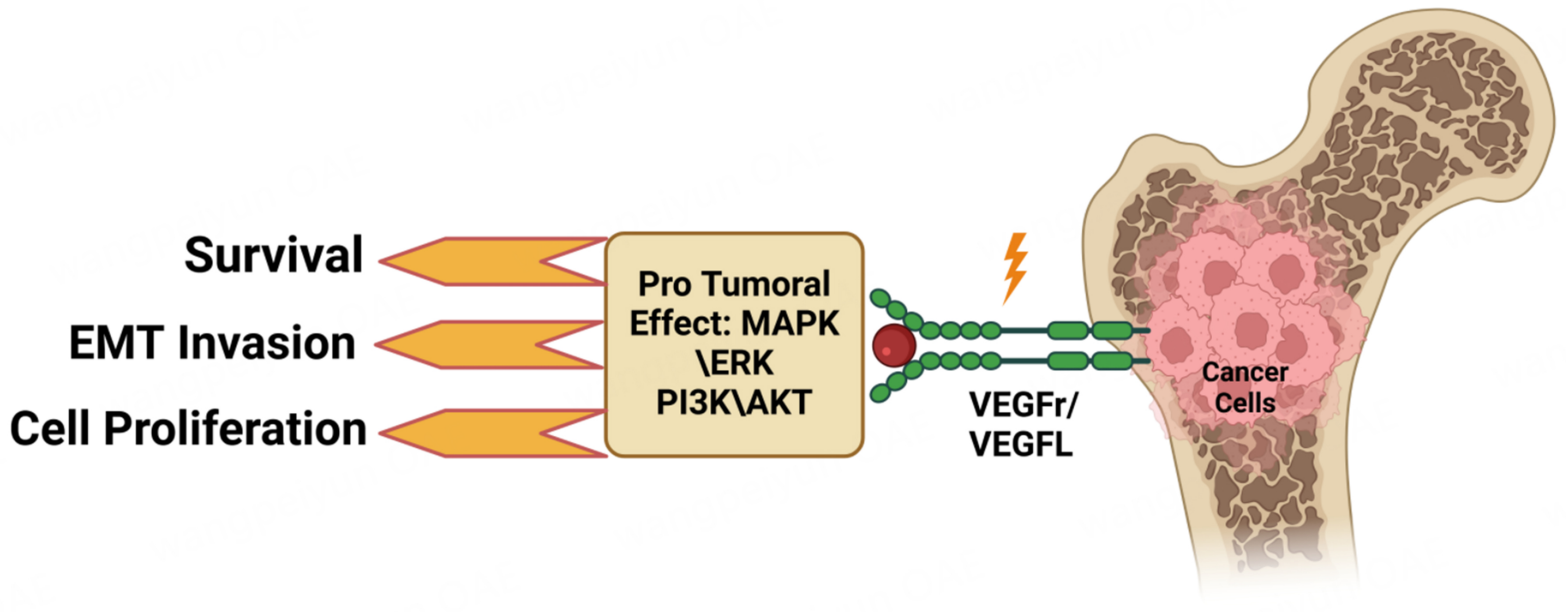
This schematic highlights the role of VEGF signaling in driving bone-metastatic tumor progression. Engagement of VEGFL with its receptor (VEGFr) on tumor cells activates downstream pro-tumoral pathways - including MAPK/ERK and PI3K/AKT - that enhance cell survival, EMT, and proliferative capacity. Collectively, these signaling cascades promote cancer cell invasion, growth, and survival within the bone microenvironment. Created in BioRender. Mohammad, K. (2025) https://BioRender.com/ig2q2za. VEGF: Vascular endothelial growth factor; VEGFL: VEGF ligand; MAPK: mitogen-activated protein kinase; EMT: epithelial-to-mesenchymal transition.

Anti-angiogenic therapies targeting VEGF/VEGFR pathways often fail in bone metastasis due to alternative pro-angiogenic mechanisms and VEGF-independent survival pathways [Figure 3]. HIF-1α promotes angiogenesis by inducing VEGF, FGF, PDGF, TGF, and Ang expression, enhancing blood vessel formation and neoplastic cell survival^[133]^.

The growth of bone and soft tissue sarcomas relies on angiogenesis, predominantly via the VEGF/VEGFR pathway [Figure 3]. Although various VEGFR inhibitors have shown promise in other cancers, they are less effective in advanced sarcomas due to drug tolerance, short response duration, and severe side effects. However, apatinib, a novel VEGFR2 inhibitor, demonstrates strong anti-angiogenic and antitumor effects, reversing multidrug resistance, promoting tumor regression, and improving chemotherapy and radiotherapy efficacy. Yet, overcoming apatinib’s resistance remains a challenge^[134]^.

### ECM remodeling

The molecular mechanisms facilitating the progression of cancer cells through these biological stages have been the subject of thorough research^[135-137]^. The reorganization of the actin cytoskeleton, alteration of the ECM, and the recruitment of specific immune cells enable effective ingress into the circulatory system. The aggregation of migratory cancer cells enveloped in platelets and metabolic reprogramming confer resistance to mechanical injury, innate immune response, anoikis, and oxidative stress in the bloodstream^[138-140]^.

Matrix metalloproteinases (MMPs) are key enzymes involved in ECM degradation, critical for bone metastasis. They influence tumor invasion, angiogenesis, and metastatic foci formation while regulating factors like RANKL and TGFβ. Understanding MMP regulation and its role in drug resistance is essential for developing effective treatments against bone metastases^[141-143]^. MMP-2 and MMP-13 have been emphasized for their involvement in bone metastasis. MMP-2 facilitates breast cancer metastasis to bone by influencing every phase of the metastatic cascade, whereas MMP-13 is modulated by the small leucine zipper protein (sLZIP) in CRPC, thereby enhancing metastasis^[141,144]^.

MMPs, especially MMP7, are associated with acquired drug resistance in cancer. The interplay between MMP7 and the Hsp90 chaperone is associated with tumor progression and drug resistance, though this connection necessitates additional investigation^[145]^. Although MMP inhibitors hold promise in cancer treatment, previous clinical trials have yielded unsatisfactory results. Nonetheless, advancements in the selective targeting and delivery of MMP inhibitors to the TME provide new hope for their application in the treatment of bone metastases^[141,146,147]^.

MMPs display dual roles as both pro-metastatic and anti-metastatic agents, contingent upon the context, thereby complicating their application as therapeutic targets^[142]^. MMP8 has different effects on cancers, with inhibitory effects in breast, skin, and oral, tongue cancers, but worsening prognosis in liver and gastric cancers^[148]^. This dual function requires a comprehensive understanding of their regulatory mechanisms across various cancer types. The advancement of effective MMP inhibitors poses a challenge owing to the complex functions of MMPs in cancer. Future research must concentrate on elucidating the precise contexts in which MMPs facilitate or impede metastasis to develop more efficacious therapeutic interventions^[146,147]^.

Although MMPS offers promising therapeutic targets, their complex functions necessitate meticulous evaluation in the formulation of MMP inhibitors. Enhancements in comprehending the genetic regulation of MMPs and their interactions within the TME are essential for addressing the difficulties related to their therapeutic targeting.

## MECHANISMS OF RESISTANCE TO TARGETED THERAPIES

### Hormone therapies

Androgen-deprivation therapy (ADT) is the standard treatment for metastatic PCa but also impacts bone biology. Treatment options are limited for castration-resistant cases, particularly those independent of androgen receptor (AR) function. Patients with non-AR-driven metastases may benefit from therapies targeting the TME. Bone cell activity was assessed via gene markers for osteoclasts (ACP5, CTSK, MMP9), osteoblasts (ALPL, BGLAP, RUNX2), and osteocytes (SOST). RUNX2-positive osteoblasts correlated with TRAP-positive osteoclasts on metastatic bone surfaces. Notably, bone cell activity inversely correlated with tumor AR activity (AR, FOXA1, HOXB13, KLK2, KLK3, NKX3-1, STEAP2, TMPRSS2) and PSA levels. Functional analysis revealed elevated BMP signaling in metastases with high bone cell activity and low AR activity. BMP4 positivity was confirmed in tumor cells of active bone formation sites. This inverse relationship between osteoblastic activity and AR activity may influence responses to AR-targeted or bone-targeted therapies, warranting further clinical investigation^[149]^.

### Immunotherapies

The PD-L1/PD-1 axis is integral to immune checkpoint therapy, demonstrating efficacy in the treatment of multiple cancers. Nonetheless, drug resistance, especially in bone metastases, continues to pose a considerable challenge. Tumors can elude immune detection by upregulating PD-L1, which interacts with PD-1 on T cells, suppressing their function and enabling tumor cells to evade immune surveillance. This process substantially enhances resistance in multiple cancers, such as those with bone metastases^[150-152]^.

The bone microenvironment in metastatic castration-resistant prostate cancer (mCRPC) exhibits heightened expression of inhibitory immune checkpoints, potentially facilitating resistance to PD-L1/PD-1 blockade^[153]^. Moreover, alterations in the tumor immune microenvironment, including elevated PD-L1 expression in drug-resistant neoplastic cells, exacerbate treatment challenges^[154,155]^. Resistance may also stem from genetic mutations and epigenetic alterations that influence antigen presentation and immune cell functionality. These modifications may result in immune evasion and diminished effectiveness of PD-1/PD-L1 inhibitors^[154,156]^.

Although PD-L1 expression serves as a potential biomarker for forecasting responses to PD-1/PD-L1 inhibitors, its predictive efficacy varies among different tumor types. Elevated PD-L1 expression is typically correlated with improved responses; however, this is not universally applicable. Elevated tumor mutation burden (TMB) serves as a potential biomarker, as it may be associated with improved responses to immunotherapy. Nonetheless, its predictive efficacy fluctuates and is frequently utilized alongside additional indicators^[156-158]^. Integrating PD-1/PD-L1 inhibitors with additional therapies, such as CTLA-4 blockade or myeloid compartment-targeting treatments, may improve efficacy and overcome resistance. This methodology is currently under investigation in mCRPC with bone metastases^[153]^. Combined CTLA-4 and PD-L1 blockade in patients with chemotherapy-naïve mCRPC is associated with increased myeloid and neutrophil immune subsets in the bone microenvironment^[152,153]^.

Inhibiting pathways that modulate PD-L1 expression, such as the COP1/c-Jun/HDAC3 axis, may diminish PD-L1 levels and enhance responses to PD-1/PD-L1 blockade in drug-resistant malignancies^[154]^. Formulating patient-specific treatment strategies informed by predictive biomarkers and resistance mechanisms can customize therapies to individual requirements, potentially enhancing outcomes and minimizing resistance^[159]^.

While the PD-L1/PD-1 axis is a promising immunotherapy target, overcoming resistance in bone metastases requires developing predictive biomarkers, understanding resistance mechanisms, and optimizing combination therapies.

### Chemotherapy and radiotherapy

Bone metastasis resistance to alkylating agents and taxanes involves complex genetic and molecular mechanisms, including gene amplifications, metabolic reprogramming, and altered gene expression. Resistance to alkylating drugs is linked to changes in cysteine, methionine, and purine metabolism, which differentiate drug-sensitive from resistant cells and offer potential therapeutic targets. Copy number variations and expression changes in genes like WWOX and CNTN5 affect metabolism and protein transport, contributing to resistance^[160]^. The NF-κB pathway, modulated by TNFAIP3, is crucial in conferring resistance to O6-alkylating agents. The activation of this pathway is associated with cell survival and resistance^[161]^.

NDRG1, modulated by the mTOR pathway, stabilizes MGMT, a crucial enzyme in the resistance to alkylating agents, especially under hypoxic conditions^[162]^. The amplification of extrachromosomal DNA (ecDNA) of genes such as ABCB1, which encodes P-glycoprotein, is a major contributor to taxane resistance. This mechanism results in enhanced drug efflux and diminished drug efficacy^[163]^. Modifications in tubulin genes, specifically TUBB3 and TUBB6, correlate with taxane resistance in breast cancer. These alterations influence microtubule dynamics, essential for taxane effectiveness^[164]^.

Taxane resistance is associated with EMT and neuroendocrine phenotypes, which correlate with enhanced cellular plasticity and resistance^[165]^. Chemotherapy activates the JNK pathway, enhancing ECM remodeling, wound healing, and stem cell networks, reducing treatment efficacy. Elevated JNK activity, linked to poor breast cancer outcomes, promotes tumor growth and metastasis by upregulating ECM components SPP1 and TNC, which are c-Jun targets. Blocking JNK or altering SPP1/TNC expression improves chemotherapy sensitivity, suggesting new strategies for treating metastatic breast cancer^[166]^.

## FUTURE DIRECTIONS

### Genomic biomarkers predicting response or resistance

Identifying genomic biomarkers for bone metastases is crucial for optimizing treatment in prostate, breast, and lung cancers. EGFR mutations in LADC are key biomarkers predicting response to EGFR TKIs, particularly in osteoblastic metastases evolving from osteolytic lesions. Additionally, KRAS mutations are associated with poor prognosis in lung cancer with bone metastases, highlighting their importance in guiding therapeutic decisions^[167]^. Serum alkaline phosphatase (ALP), which has been shown to correlate with bone metastasis activity and treatment response, is another important marker for bone metastases. Elevated levels of ALP prior to treatment with Radium-223 have been associated with worse outcomes, indicating its potential role as a prognostic and response biomarker in mCRPC^[168]^. Similarly, studies have indicated that serum calcium levels can serve as valuable biomarkers for early diagnosis of bone metastases in cancer patients, including those with lung cancer^[169]^.

### Advances in genomic technologies

Single-cell sequencing and CRISPR technologies are essential for uncovering resistance mechanisms in bone metastases. Single-cell sequencing reveals tumor heterogeneity, identifying resistant subpopulations with distinct transcriptional profiles that enhance survival in the bone microenvironment. Disseminated tumor cells (DTCs) in bone marrow often display stemness markers and apoptosis resistance, suggesting that the bone niche supports their survival and metastatic potential^[170,171]^. Moreover, studies have shown that hybrid cells in the bone marrow, which arise from the fusion of tumor and immune cells, can promote a more immunosuppressive microenvironment, further complicating treatment responses^[172]^. This highlights the importance of understanding the interactions between tumor cells and their microenvironment, as these interactions can significantly influence metastatic behavior and therapeutic resistance.

CRISPR technology complements single-cell sequencing by enabling precise genetic modifications to study the functional roles of specific genes in resistance mechanisms. For example, CRISPR can be employed to knock out genes implicated in the survival of cancer cells within the bone microenvironment, allowing researchers to assess the impact of these genes on metastatic outgrowth and resistance to therapies^[173]^. Additionally, CRISPR-based screens can identify genetic vulnerabilities in resistant cancer cells, paving the way for targeted therapies that exploit these weaknesses^[174]^. The ability to manipulate the genome of cancer cells *in vivo* has opened new avenues for understanding how specific genetic alterations contribute to the metastatic process and resistance to treatment.

Combining single-cell sequencing with CRISPR enables detailed analysis of cell interactions within the metastatic bone niche. This approach reveals how cells like osteocytes and macrophages support metastasis and drug resistance. For example, single-cell studies have identified senescent osteocytes that secrete bone-destructive factors enhancing breast cancer metastasis^[175]^. CRISPR can target these pathways, offering potential therapeutic strategies to counter bone metastasis.

### Challenges in translational research

Genomic profiling of bone metastases faces major challenges due to biological and technical barriers. These metastases, common in advanced breast and PCas, are hard to access and analyze because of their complex microenvironment and limitations in imaging and sampling. Obtaining representative samples is difficult, especially in fibrotic bone marrow, where bone marrow aspirates often result in non-informative “dry taps”^[176]^. This limitation hinders comprehensive genomic profiling, as samples may not accurately represent the metastatic tumor’s genetic landscape. Alternatives like FISH and microarray-based profiling on bone marrow biopsies have been proposed, but they still face issues related to sample quality and accessibility^[176]^.

The inherent biological characteristics of bone metastases complicate genomic profiling efforts. The bone microenvironment is not merely a passive site for tumor growth; it actively influences tumor behavior through complex interactions between cancer cells and the surrounding stromal cells^[177,178]^. This interaction can lead to therapeutic resistance and complicate the identification of actionable genomic alterations. For instance, the osteogenic niche within the bone serves as a reservoir for calcium and other factors that can affect tumor cell survival and proliferation, thereby impacting the efficacy of treatments targeting these metastases^[179]^. Understanding these interactions is crucial for developing effective genomic profiling strategies that can inform therapeutic decisions.

Additionally, the limitations of current imaging modalities further exacerbate the challenges associated with accessing bone metastases. While advanced imaging techniques such as positron emission tomography (PET) and magnetic resonance imaging (MRI) are becoming more prevalent, they still face limitations in sensitivity and specificity for detecting bone metastases^[180,181]^. Conventional imaging often provides indirect markers of cancer activity, which may not accurately reflect the genomic characteristics of the tumor^[180]^. The integration of multimodal imaging approaches has shown promise in enhancing the detection of bone metastases, yet the translation of these techniques into clinical practice remains a hurdle^[182]^.

### Development of novel therapies

Targeting genetic vulnerabilities in drug-resistant bone metastases represents a promising therapeutic strategy, particularly in the context of prostate and breast cancers. Current treatments for bone metastases, such as bisphosphonates and denosumab, primarily alleviate symptoms and prevent skeletal-related events (SREs) but do not significantly enhance overall survival rates in patients with advanced cancers^[48,183]^. Therefore, innovative approaches that focus on the underlying genetic and molecular mechanisms driving drug resistance are urgently needed.

One of the critical pathways implicated in the development of drug-resistant bone metastases is the TGFβ/Ac-KLF5 axis. Research has shown that TGFβ induces acetylation of KLF5, which promotes EMT and tumorigenicity, thereby enhancing chemoresistance in PCa^[49]^. Targeting acetylated KLF5 could improve the specificity of therapies that inhibit TGF-β signaling, which has been a challenge due to the widespread distribution of TGFβ in various tissues^[49]^. This specificity is crucial, as non-specific targeting can lead to adverse effects and limit the therapeutic efficacy of TGFβ inhibitors.

The insulin-like growth factor (IGF) axis has been identified as a significant player in the progression of bone metastases. IGFs contribute to the hallmarks of cancer, including treatment resistance, by influencing stem cell renewal and differentiation processes^[184]^. Therefore, therapies that target the IGF signaling pathway may offer a dual benefit: inhibiting tumor growth and overcoming resistance mechanisms. Additionally, the role of growth differentiation factor 15 (GDF15) has emerged as a biomarker associated with poor prognosis and drug resistance in various malignancies, including lung cancer with bone metastases^[185]^. Elevated GDF15 levels correlate with increased metastatic potential and resistance to therapies, indicating that targeting this factor could enhance treatment outcomes.

Nanomedicine also presents a novel approach to improve drug delivery to bone metastases. Traditional chemotherapeutics often fail to achieve therapeutic concentrations in bone due to low blood flow and the unique microenvironment of bone tissue^[186]^. Nanoparticles designed to target bone metastases can enhance drug delivery while minimizing systemic side effects, thus improving therapeutic efficacy^[186]^. For instance, stem cell membrane-coated nanoparticles have shown promise in delivering chemotherapy and immunotherapy agents directly to bone metastases, potentially overcoming the challenges posed by the TME^[187]^.

## CONCLUSION

In conclusion, genetic and epigenetic alterations are pivotal in driving drug resistance in bone metastases. Mutations in key driver genes and epigenetic changes shape the complex resistance landscape, while the unique bone microenvironment acts as a protective niche for cancer cells. Advances in genomic and epigenetic profiling underscore the need for personalized, targeted therapies, including CRISPR-based gene editing and epigenetic modulators. Integrating these innovations with conventional treatments could transform bone metastasis management.

A multidisciplinary approach, combining genomic insights, molecular biology, and clinical oncology, is essential to overcoming therapeutic resistance. Continued research and collaboration will be vital to improving treatment outcomes and minimizing patient burden.

## DECLARATIONS

### Copyright

© The Author(s) 2025.
